# Psychological Disorders and Ecological Factors Affect the Development of Executive Functions: Some Perspectives

**DOI:** 10.3389/fpsyt.2016.00195

**Published:** 2016-12-07

**Authors:** Rafika Zebdi, Louise Goyet, Charlotte Pinabiaux, Bahia Guellaï

**Affiliations:** ^1^Laboratoire EvaCliPsy (EA CLIPSYD 4430), Université Paris Ouest Nanterre La Défense, Nanterre, France; ^2^Laboratoire Paragraphe (EA 349), Université Paris 8 Vincennes-Saint-Denis, Saint-Denis, France; ^3^Laboratoire CHArt (EA 4004), Université Paris Ouest Nanterre La Défense, Nanterre, France; ^4^Laboratoire Ethologie, Cognition, Développement, Université Paris Ouest Nanterre La Défense, Nanterre, France

**Keywords:** psychological disorders, development, parenting, executive functions, children

## Abstract

The links between deficits in executive functions (EFs) (e.g., mental flexibility, inhibition capacities, etc.) and some psychological disorders (e.g., anxiety and depressive disorders) have been investigated in the past decades or so. Observations evidenced that some deficits in working memory, planning, and mental flexibility were highly correlated with anxiety and depressive disorders. The majority of studies focused on adults’ population, whereas it seems important to adopt a developmental perspective to fully understand the dynamic relation of these EF/psychological disorders. We suggest to focus on the following two axes in future research: (i) relations between EF and anxiety traits through development and (ii) the possible role of external factors such as parent–child relationships on the development of EF.

## Introduction

One possible short definition of executive functions (EFs) is that they are adaptive, goal-directed behaviors that enable individuals to override more automatic responses (1, 2). EFs reflect a cognitive function that incorporates a set of abilities, which aims to coordinate and control processes such as planning skills. From a developmental perspective, EFs play an important role in the development of learning and in socio-cognitive abilities (3, 4). Although the study of EF in adulthood has long been a field of active research, little is known about early EF development. One of the reasons for this gap is the lack of age-appropriate EF tasks. Nonetheless, some studies evidenced that the first 5 years of life play a critical role in the development of EF (5, 6). Early work on infants and primates suggests that the prefrontal cortex (PFC) is operative since birth in humans and in other species (7–11). In fact, children are born with the potential to develop these skills. Despite growing interest in the development of EF, it is not clear whether this development is associated with psychological factors and/or external ones. Indeed, some clinical studies evidenced that EF deficits can be impaired by specific psychological factors such as anxiety depression and stress in adults (12, 13), whereas other studies evidenced that ecological factors such as socioeconomic status (SES) could impact EF.

In the present paper, we propose to explore the possible links between the development of EF, psychological disorders, and ecological factors based on the following two axes: (i) the links between EF and psychological factors such as anxiety disorders and (ii) the possible role of ecological factors such as macro- and micro-environmental characteristics on the development of EF.

## Relations Between Executive Functions and Anxiety Disorders

In this section, we present how psychological disorders such as anxiety can affect the development of EF in adults and adolescents. The links between EF and anxiety are more and more explored since the last decades or so (14–17). Nonetheless, the relations between EF and anxiety from a developmental perspective are far from being established and clear. Still, we do not know much about the developmental trajectory of this link and the factors that could play a role.

Deficits in some aspects of EF such as attention and memory are frequently reported in studies on anxiety disorders in adults. The main anxiety disorders explored in these studies are obsessive–compulsive disorder (OCD), posttraumatic stress disorder (PTSD), social anxiety disorder (SAD), generalized anxiety disorder (GAD), and panic disorder at a given age (children, teenagers, or adults).

Goussé et al. (18) found that adults with OCD symptoms have specific deficits in spatial working memory and planning. Memory, attention, and processing speed are also impaired in these adult populations (19). The dysfunction of EF, attention, verbal, and visual memory has been reported as being associated with PTSD in adults (20). Olff et al. (21) also indicate that PTSD patients have poorer flexibility and set shifting, planning, and working memory than trauma-exposed controls. Other studies have also shown deficits in EF in patients with SAD, such as poorer cognitive flexibility (22, 23). There are also links between panic disorders and deficits in EF in adults (24).

These relations between anxiety disorders and deficits in EF in adults can be in part explained by external factors. Indeed, stressful life events can cause temporary changes in PFC functioning, which negatively affects EF and results in impaired ability for cognitive inhibition (25).

Interestingly, the relations between anxiety disorders and EF impairment are present earlier than adulthood, but results are mixed. For example, teenagers (i.e., a non-clinical population), who present anxiety symptoms such as GAD, have been reported with deficits in EF in some studies (26). A study by Yang et al. (27) showed that children and adolescents with PTSD related to a natural disaster have deficits only in the emotional control domain compared with controls exposed to the same disaster. Other studies evidenced no links between GAD and EF in clinical adults’ samples (14, 28). How can we explain those mixed results? One way would be to take a developmental perspective. For example, children with OCD symptoms showed deficits in mental set shifting supporting the frontal–striatal dysfunction hypothesis of OCD diagnosis in children as well as in adults (29). Van den Heuvel et al.’s (30) study supports the hypothesis that decreased dorsal prefrontal–striatal responsiveness is associated with impaired planning capacity in OCD patients. Because the described frontal–striatal dysfunction in OCD is independent of state anxiety and disease symptom severity, they concluded that executive impairment is a core feature in OCD.

Other studies exist but they considered a broader range of disorders called “internalizing problems” that encompass anxiety disorders and depression. Riggs et al. (31) conducted a prospective study with 6- to 9-year-old children in regular classrooms over a period of 2 years. They found that proficient inhibitory control and sequencing ability were predictive of reduction of internalizing problems. Besides, some studies with children and adolescents with internalizing disorders have evidenced impaired performance on several aspects of the EF especially linked to anxiety profiles (29, 32, 33). For example, children with OCD have impaired perceptual organization ability under time pressure compared to a control population.

Longitudinal studies are needed to explore the developmental aspect of EF, but their existence is very sparse so far. Indeed, such studies demand a very large data collection with large samples and a lot of time for delayed results. Besides, in this particular area, the lack of developmental studies may be also explained by two factors: (1) late interest in studying the link between EF and psychological disorders through development trajectories (4) and (2) differences of conceptual models of anxiety disorders that are not easily understandable from a neuropsychological perspective (17). Until now the psychological models of anxiety have only been less integrated to neuroscience research (17, 34–36). Theoretical progress is necessary to fill the gap between neurobiological data, self-assessment, and clinical data. Sharp et al. (17) suggested the creation of transdiagnostics anxiety constructs that are particularly useful to connect the psychophysiological data and the psychological models of anxiety. More precisely, some studies evidenced both heightened right-lateralized activity and left-lateralized activity across a wide range of DSM anxiety disorders such as GAD, panic disorder, SAD, and OCD (37, 38). It is an important point as authors (17) suggest to disentangle between the anxious apprehension and the anxious arousal during the examination of anxiety with regard to other constructs such as EF to avoid confusion (39).

## The Possible Role of Ecological Factors on the Development of EF

Based on Bronfenbrenner’s ecological systems theory (40), it might be hypothesized that some external factors could play a role in the development of EF. This hypothesis identifies some environmental factors with which an individual interacts, such as the macrosystem that refers to the cultural contexts, or SES in which individuals live, and the microsystem that describes the groups that directly influence the child’s development (e.g., family members and language). Thus, in this section, we review possible links between the development of EF and some macro- and micro-environmental factors. Given its physiological characteristics such as longer maturation, the PFC is especially sensitive to ecological factors (41, 42). Therefore, EFs are thought to develop as a result of a dynamic interaction between the child’s PFC and the external environment (43, 44). The link between characteristics of the child’s environment, as well as the quality of parent–child relationships, and child’s cognitive development has received growing interest in the past decades or so (45).

Ecological influences may be conceptualized at different levels including the macro-environments (i.e., cultural context, for example, SES), and the micro-environments (i.e., the family setting and parent–child relationships) (46, 47). Thus, microenvironmental factors such as parents “scaffolding” (48) means that a child reach higher levels of comprehension and skill acquisition, thanks to adults’ support. At the beginning, they are dependent on adult support, and then they become more independent of the way they acquire new knowledge. Thus, this support could help children to improve their executive function skills (learning to coordinate and control processes) before they must perform by their own. It is important for children to develop these skills through social relations. Indeed, social partners will teach them to cope with stress, to face issues, and to provide opportunities for directing their own actions (decision making) without any adult’s control. It was shown that adverse environments resulting from neglect, abuse, violence, stress, and SES of the family may expose children to toxic stress, which disrupts brain architecture and impairs and seriously delays the development of EF (49, 50). Also, stress, lack of sleep, loneliness, or lack of exercise each could impair EF (51).

### Macro-Environmental Factors and the Development of EF

An important macro-environmental factor that seems to play a role on the development of EF is the characteristics of the family setting such as SES. Some studies demonstrated that family SES is associated with children’s working memory and cognitive control (52–55). More recently, Sarsour et al. (56) studied the independent and interactive associations between family SES and single parenthood to predict child EF. Single parent and family SES were associated with children’ inhibitory control and cognitive flexibility such that children from low SES families who were living with one parent performed less well on EF tests than children from similarly low SES who were living with two parents. Interestingly, this study demonstrates interactions between different external factors and children EF. Moreover, mediation models help us to better understand the interaction between macro-environmental and the development of EF: the links between SES and EF are at least partially explained by associated variations in parenting behaviors (57). Besides, the level of stress, associated with a negative affectivity, has also been pointed out for its mediation role between macro-environmental factors and the development of EF (58).

### Micro-Environmental Factors and the Development of EF

#### The Case of Bilingualism

An important aspect in the child’s micro-environment is the language used at home and more specifically the fact that children may grow up in a mono- or bi-lingual environment. Some studies evidenced enhanced abilities of bilingual children to coordinate the executive control components required in performing complex tasks (59, 60). This link appears to be present early in the development as an advantage for bilingual 7- to 12-month-old infants on inhibitory control and attention over monolingual infants of the same age has been evidenced (61, 62). Therefore, it seems that executive control develops earlier in bilingual children than in comparable monolinguals (63, 64). This advantage continues to be present later in the development as bilingual adults still outperform monolinguals on EF tasks (65–67). Therefore, the mastery of two languages provides bilingual speakers cognitive benefits over monolinguals, particularly on cognitive flexibility and selective attention. To the best of our knowledge, only one study explored the effect of the bilingualism factor on EF longitudinally showing a task-specific advantage in inhibitory control in bilingual toddlers (68). Another micro-environmental factor that influences the development of EF is the role of parenting.

#### The Role of Parenting

Vygotsky (69) accounted that the interaction with a more competent social partner, such as the parent, fosters children’s higher order cognitive functions. From this socio-constructivist point of view, the development of EF can be considered as a transfer from inter- to intra-personal regulation. The link between quality of parent–infant interactions and subsequent child’s EF has been explored only recently.

Three dimensions of parenting have been associated with the development of EF (70): scaffolding, sensitivity, and mind-mindedness. Most of the studies have focused on scaffolding. This large concept refers to how parental guidance enables children to achieve level of problem solving, which they could not have reached on their own. Hughes and Ensor (71) showed that maternal scaffolding was more predictable of the development of EF at 2–4 years than imitation model or than more global positive or negative models. In another study, Bernier et al. (72) explored the relationship between scaffolding (measured as autonomy support), sensitivity, and mind-mindedness on the one hand and conflict EF^1^ and impulse control on the other hand. Parenting habits were observed during free play and problem solving sessions at 12, 18, and 23 months and were correlated with several measures of EF at 18 and 26 months in 80 mother–child dyads. Independent of maternal education and general cognitive ability, they found that (1) sensitivity at 12 months was predictive of the development of conflict EF at 26 months; (2) mind-mindedness at 12 months was predictive of working memory at 18 months and of increase in EF skills between 18 and 23 months; and (3) scaffolding at 12 months was predictive of working memory and categorization at 18 months and conflict EF at 26 months. In another study, Bernier et al. (73) investigated the link between maternal interactive behavior, paternal interactive behavior, and child attachment security between 1 and 2 years of age, and child EF at 2 and 3 years. The results suggest that parental behavior and child attachment are related to child performance on EF tasks especially on cognitive flexibility components. These findings suggest that parent–child relationships may play an important role in children’s developing self-regulatory capacities. More recently, Bernier et al. (74) confirmed this predictive role of attachment security at 2 years on the development of EF at 5–6 years.

The following two explanations can be made: (1) parenting provides the child with the social context in which to practice emerging regulatory skills and (2) parenting may affect brain structures involved in EF, especially the response to stress system, as it has been demonstrated in studies with animals (75). Indeed, Wagner et al. (76) evidenced that children with poorer EF had higher levels of salivary cortisol (i.e., related to stress level), and their parents reported higher parenting stress. Hence it seems that parenting and other psychological factors such as stress are important to understand the development of EF.

## Conclusion

The idea of the present review was to explore the relations between EF and factors such as anxiety disorders and parenting through development. Much of the early influences on later EF dysfunctions appear to be transmitted through the quality of parent–child interaction during early childhood (57) and may also depend on the development of frontal brain areas and the stress response system. Interestingly, the studies reporting relationships between parenting practice and the development of EF have pointed out impacts only on some specific aspects of EF, namely, working memory and cognitive flexibility (71–73, 77). Possible hypothesis can be proposed to explain the link between EF, anxiety, and some ecological factors: (1) parenting is the main factor that impact EF development; (2) SES and bilingual environments are two important side factors playing a role in the development of EF as they are related to parental practices; and (3) poorer parenting behaviors together with a poor environment could impact EF development and predict anxiety symptoms. Interestingly, these possible scenarios are linked together. Future studies should explore the neural and social mechanisms underlying the links between parent–child relationships and the development of EF. Here, we also highlight the links between some anxiety disorders and deficits in EF. Future clinical research should take into account the transdiagnosic anxiety construct (17). It is possible that different levels of anxiety apprehension and anxiety arousal could explain results observed in previous research on the link between anxiety and EF. If these two dimensions are taken into account in a transdiagnosis construct, we could precise which dimension is associated with which aspect of EF independent of the DSM diagnosis. Investigating this axis of research would also help clarifying the pathogenesis of diverse forms of anxiety and the potential deficits in EF through development. In that sense, it would be interesting to explore the multifactorial dimensions (i.e., internal and external factors: anxiety and parent–child relationships) on the development of EF. Innovative tools, providing the support for children to develop these EF skills at home, in early care units, and in education programs, will offer new perspectives to explore the influence of children environment, parenting behavior, and clinical profiles on early development of EF. Furthermore, if we have to take into account the developmental timetable, ecological and external factors, and the variability of the clinical symptomatology, new studies should investigate the development of EF from a longitudinal perspective with infants’ population and the impact of this factors on EF (cognitive) development. These new directions of research could also help to promote clinical and neuropsychological tests and to set up remediation tools for very young children.

## Author Contributions

All the authors participated to writing of the paper. RZ and BG contributed equally to the writing.

## Conflict of Interest Statement

The authors declare that the research was conducted in the absence of any commercial or financial relationships that could be construed as a potential conflict of interest.

## Footnotes

^1^Factors including measures of working memory, set shifting, and inhibition control.
